# Bone Marrow Derived Mesenchymal Stromal Cells Ameliorate Ischemia/Reperfusion Injury-Induced Acute Kidney Injury in Rats via Secreting Tumor Necrosis Factor-Inducible Gene 6 Protein

**DOI:** 10.1155/2019/9845709

**Published:** 2019-03-11

**Authors:** Yue Chen, Xiaochen Tang, Ping Li, Ying Zhou, Ting Xue, Jie Liu, Chen Yu

**Affiliations:** ^1^Department of Nephrology, Tongji Hospital, Tongji University School of Medicine, Shanghai 200065, China; ^2^Translational Center for Stem Cell Research, Tongji Hospital, Stem Cell Research Center, Tongji University School of Medicine, Shanghai 200065, China; ^3^School of Life Sciences, Fudan University, Shanghai 200433, China; ^4^Department of Hematology, Tongji Hospital, Tongji University School of Medicine, Shanghai 200065, China

## Abstract

**Aims:**

To investigate whether bone marrow derived mesenchymal stromal cells (BMSC) have ameliorated ischemia/reperfusion injury-induced acute kidney injury (IRI-AKI) via tumor necrosis factor-inducible gene 6 protein (TSG-6) and how TSG-6 exerted this effect.

**Methods:**

We used lentiviral vectors of short hairpin RNA (shRNA) targeting TSG-6 gene to silence TSG-6 in BMSC. And TSG-6-silenced BMSC were administrated into IRI-AKI rats. Then we analyzed serum creatinine (Scr) and renal histology of IRI-AKI rats treated with BMSC after different pretreatments. Furthermore, we explored the effect of TSG-6 on renal tubular epithelial cells proliferation* in vivo* and* in vitro* assays.

**Results:**

The Scr levels of IRI-AKI rats treated with BMSC (73.5±7.8 *μ*mol/L) significantly decreased compared to those of IRI-AKI rats treated without BMSC (392.5±24.8 *μ*mol/L, P<0.05) or with DMEM (314.0±19.8 *μ*mol/L, P<0.05). Meanwhile, the renal tissue injury in IRI-AKI rats treated with BMSC improved markedly. However, the Scr levels of IRI-AKI rats treated with TSG-6-silenced BMSC (265.1±21.2 *μ*mol/L) significantly increased compared to those with BMSC (74.0±8.5 *μ*mol/L, P<0.05). The proportion of Ki67-positive cells was reduced in IRI-AKI rats treated with TSG-6-silenced BMSC compared to that in IRI-AKI rats treated with BMSC (29.7±0.8% versus 43.4±3.0%, P<0.05).* In vitro*, the cell proliferation rate of TSG-6-stimulated NRK-52E cells under hypoxia (89.2±3.9%) increased significantly compared to that of NRK-52E cells alone under hypoxia (82.4±0.8%, P<0.05). Similarly, the proportion of Ki67-positive cells was significantly elevated in TSG-6-stimulated NRK-52E cells under hypoxia. Furthermore, TSG-6 could inhibit infiltration of neutrophils in kidney tissue of IRI-AKI.

**Conclusions:**

TSG-6 plays a key role in the treatment of IRI-AKI with BMSC, which may be due to its effect on promoting renal tubular epithelial cells proliferation by modulating inflammation.

## 1. Introduction

Acute kidney injury (AKI) is a common clinical syndrome characterized by renal dysfunction, which is caused by a spectrum of etiologies in multiple settings. AKI occurs in about 13.3 million people per year, 85% of whom live in the developing world. Moreover, AKI is thought to contribute to about 1.7 million deaths every year [1]. In China, the epidemiological data shows that at least 2.9 million people suffer from AKI, which need to be treated in hospital each year, and 0.7 million of them die. About two-thirds of the survivors will develop chronic kidney disease (CKD) [2]. AKI is the main contributory factor for poor prognosis, which not only increases the incidence of CKD and causes end-stage renal disease but also increases mortality in critically ill patients [3]. AKI has become a global health hot topic. However, the treatment options for patients with AKI are limited, mainly including renal replacement therapy or waiting for renal self-repair.

The pathological changes of AKI are characterized by renal tubular injury and renal interstitial inflammation [4]. The main etiologies of AKI include ischemia/reperfusion injury (IRI), nephrotoxic drugs, and sepsis. The damaged renal tubular epithelial cells participate in the chemotaxis and activation of inflammatory cells by releasing cytokines, which further amplifies the inflammatory response in the kidney. Meanwhile, the inflammatory immune response plays an important part in the process of acute kidney injury and repair. Although the damaged renal tubular epithelial cells have the ability to repair and regenerate, this ability cannot meet the urgent need for renal recovery in most cases. Therefore, it has become a research focus to promote AKI repair in the field of nephrology.

In recent years, stem cell therapy has become a novel cell-based therapy for several inflammatory diseases. Experimental studies have shown that mesenchymal stromal cells (MSC) can promote AKI repair [5]. However, the research on MSC ameliorating AKI is mainly based on experimental animals. The clinical application of MSC is limited due to their unclear mechanisms. Several studies have suggested that MSC exert their effect on AKI via the paracrine mechanism [6]. In the damaged microenvironment, activated MSC can secrete a variety of anti-inflammatory factors to promote tissue repair, including tumor necrosis factor-inducible gene 6 protein (TSG-6), prostaglandin E2, and interleukin-1 receptor antagonist [7]. Among them, TSG-6 has displayed remarkable therapeutic effects in several models of acute organs injury [7]. However, there are very few studies on the effect of TSG-6 secreted by MSC in AKI.

In view of TSG-6 as a protective inflammatory response gene, we hypothesized that bone marrow derived MSC (BMSC) might exert their therapeutic effect by secreting TSG-6 in AKI. Then we tested our hypothesis in a series of* in vivo* and* in vitro* assays. The effect of TSG-6 was verified by administrating TSG-6 silenced-BMSC into IRI-induced AKI (IRI-AKI) rats and was then examined in rat renal tubular epithelial cells under hypoxia. Our results verified that TSG-6 was the key factor that allowed BMSC to treat IRI-AKI and we also discussed its possible mechanism.

## 2. Materials and Methods

### 2.1. Procedure and Protocol of IRI-AKI

Pathogen-free, adult male Sprague-Dawley (SD) rats (Shanghai Laboratory Animal Research Center, Shanghai, China) weighing 200±10 g were utilized in the present study. The protocol for the acute kidney ischemia/reperfusion procedure has been detailed in our previous reports [8]. Briefly, animals were anesthetized by sodium pentobarbital (40 mg/kg, intraperitoneally) and placed on a warming pad to maintain body temperature at 37°С for midline laparotomies. The sham control animals underwent laparotomy only. Acute IRI of both kidneys was induced in all IRI-AKI rats by clamping the renal pedicles for 45 min using nontraumatic vascular clips.

### 2.2. Rat BMSC Isolation and Identification

Rat BMSC were isolated and harvested as follows. Briefly, 3- to 4-week-old SD rats were sacrificed and soaked in 75% alcohol for 10 min. Under aseptic conditions, the femurs and tibias of SD rats were taken out and flushed with phosphate-buffered saline (PBS). By rinsing the bone marrow cavity, cell suspension was collected and cultured in 60 mm culture dish at 37°C in a humidified atmosphere of 5% CO_2_. The cell culture medium was Dulbecco modified Eagle's medium (DMEM) (Gibco, NY, USA) supplemented with 10% fetal bovine serum (FBS, Gibco, NY, USA). The nonadherent cells were removed every 2 days and primary adherent cells were subcultivated 1:2 until the cells reached around 80% confluence. The typical markers (CD29, CD44, and CD90) of BMSC were detected in the cells of passage 3 by flow cytometry. Also, the cells were tested for their ability to differentiate into adipogenic, chondrogenic, and osteogenic lineages by a manufacturer of differentiation kits, including StemPro™ Adipogenesis Differentiation Kit (A1007001, Gibco, NY, USA), StemPro™ Chondrogenesis Differentiation Kit (A1007101, Gibco, NY, USA), and StemPro™ Osteogenesis Differentiation Kit (A1007201, Gibco, NY, USA). The BMSC of passages 3-5 were used in animal experiments.

### 2.3. NRK-52E Cells Culture and Grouping

NRK-52E cells, which were rat renal tubular epithelial cell line, were purchased from the cell bank of Chinese Academy of Sciences (Shanghai, China). Cells were cultured in DMEM (Gibco, NY, USA) supplemented with 5% FBS (Gibco, NY, USA). Cells were grown at 37°C in a humidified atmosphere with 5% CO_2_ and changed with fresh growth medium every 2 days until confluence. Cells were isolated by trypsinization when near confluence. Serum-free medium with 150*μ*M cobalt dichloride (CoCl_2_) was added to mimic hypoxic conditions [9]. The NRK-52E cells were divided into three groups (A, B, and C). In group A: control normoxic cells were maintained in normal atmosphere; in group B: serum-free medium with 150*μ*M CoCl_2_ was added for a 48 h incubation after normoxic culture; in group C: serum-free medium with 150*μ*M CoCl_2_ and 0.1*μ*g/ml recombinant human TSG-6 (rTSG-6, 2104-TS-050, R&D Systems) were added for a 48 h incubation after normoxic culture. Before the stimulation experiments, cells were growth-arrested in DMEM without FBS for 24 h. Each group was established in three holes and cultured in 6 times repeatedly.

### 2.4. Lentiviral Vectors of TSG-6 shRNA Silencing TSG-6 in Cells

For RNA interference experiments, short hairpin RNA (shRNA) targeting rat TSG-6 gene (GenBank accession number: NM_053382.1) (TSG-6 shRNA) was designed and synthetized. Meanwhile, the scrambled control shRNA (sc-shRNA) was designed and synthetized. The sc-shRNA was nontargeting shRNA, which was demonstrated to have no homology to rat genes. The forward sequences of DNA corresponding to TSG-6 shRNAs and sc-shRNA were as follows:

TSG-6-1:

  GATCCGCAGCAGGCGTATACCATAGATTCAAGAGATCTATGGTATACGCCTGCTGCTTTTTTGGTACCG;

TSG-6-2:

  GATCCGGCAGATACAAGCTAACCTATTTCAAGAGAATAGGTTAGCTTGTATCTGCCTTTTTTGGTACCG;

Scrambled control:

  GATCCAGCAGCATTAGTCGAACCGAGTTCAAGAGACTCGGTTCGACTAATGCTGCTTTTTTTGGTACCG.

The lentiviral vectors of TSG-6 shRNAs were constructed by iCarTab Biomedical Inc. (Suzhou, China). The lentiviral vectors had reporter gene (green fluorescent protein, GFP). BMSC were transfected with TSG-6 shRNA or sc-shRNA with a commercial kit (iCarTab Biomedical Inc., Suzhou, China). Briefly, BMSC were seeded into 150 cm^2^ flasks at the density of 3 × 10^6^/flask. Then, complete culture medium was added and cells were cultured overnight. Next, 100 *μ*l lentivirus was added to the 150 cm^2^ flasks and blended with the pipette gently. The flasks were centrifuged at the speed of 800 g for 1 h. After that, the BMSC in flasks continued to be cultured in the incubator for 48 h. Under laser confocal microscope, the efficiencies of transfecting TSG-6 shRNA into BMSC were assessed. At last, shRNA-silenced BMSC were collected and total RNA was extracted. Quantitative real-time PCR was used to analyze the silencing efficiencies of TSG-6 in BMSC. The primer sequences of TSG-6 and GAPDH from the cDNA of BMSC in rats were as follows: 

**Table:** 

GAPDH	sense	ACAGCAACAGGGTGGTGGAC
GAPDH	antisense	TTTGAGGGTGCAGCGAACTT
TSG-6	sense	CCACGGCTTTGTAGGAAGATAC
TSG-6	antisense	GACGCATCACTCAGAAACTTCA

TSG-6-silenced BMSC and BMSC transfected with sc-shRNA were passaged and cultured for animal experiments. Passages 3~5 cells were used for the subsequent experiments.

### 2.5. Animal Grouping and Rationale for the Therapeutic Regimen

The animals were equally categorized into groups (n=6/group): (1) sham group: laparotomy only without ischemia of bilateral renal pedicles; (2) IRI-AKI group: IRI-AKI rats without treatments; (3) DMEM group: IRI-AKI rats receiving 0.5 ml fresh DMEM by intravenous injection after ischemia; (4) BMSC group: IRI-AKI rats receiving the treatment of 5 × 10^6^ [10] BMSC by intravenous injection after ischemia; (5) sc-shRNA BMSC group: IRI-AKI rats receiving the treatment of 5 × 10^6^ BMSC transfected with sc-shRNA by intravenous injection after ischemia; (6) TSG-6 shRNA BMSC group: IRI-AKI rats receiving the treatment of 5 × 10^6^ BMSC transfected with TSG-6 shRNA by intravenous injection after ischemia. Before injection, the BMSC were resuspended in 0.5 ml fresh DMEM. The kidneys were harvested at the designated time after the surgery. Blood was collected from the inferior vena cava for measurements of serum creatinine (Scr). Scr was determined quantitatively with picric acid method on Beckman Automatic Chemistry Analyzers according to the manufacturer's instructions. All reagents used were supplied by Beckman Inc.

### 2.6. Kidney Injury Scores after IRI-AKI

Kidney injury scoring was evaluated in a blinded fashion. Briefly, the kidney tissues from experimental animals were fixed in 10% buffered formalin, embedded in paraffin, sectioned at 4 *μ*m, and stained with hematoxylin and eosin (H&E) for light microscopy. The scoring system reflects the grading of tubular necrosis, loss of brush border, cast formation, and tubular dilatation in 10 randomly chosen, nonoverlapping fields (200×) as follows: 0 (none), 1 (≤10%), 2 (11-25%), 3 (26-45%), 4 (46-75%), and 5 (≥76%) [11].

### 2.7. Immunohistochemistry and Immunocytochemistry

Immunohistochemical staining was processed in 4 *μ*m paraffinized sections. Briefly, the sections were deparaffinized. Then, 3% H_2_O_2_ was used to block endogenous peroxidase activity for 10 min at room temperature. And antigen retrieval was enhanced by microwave irradiation in citrate buffer. Nonspecific adsorption was minimized by incubating sections in normal goat serum in PBS for 20 min. Sections were incubated with the primary antibodies overnight at 4°C. The primary antibodies included rabbit anti-Ki67 (ab9260, Abcam, dilution1:100) and rabbit polyclonal to myeloperoxidase (MPO, ab45977, Abcam, dilution1:50). Then, Elivision™ plus polymer HRP (Mouse/Rabbit) IHC kit (Kit-9902, Maixin Biotechnology Corp., Fuzhou, China) was used. The sections were incubated with universal mouse/rabbit polymers for 30 min. The color reaction was developed with 3,3′-diaminobenzidine and sections were counterstained with hematoxylin. The slides were assessed by an experienced renal pathologist who knew nothing about the origin of the slides. Digital photomicrograph analysis by Image-Pro Plus 5.02 (Media Cybernetics, Silver Spring, MD, USA) was chosen to quantitate the expression of MPO in each group.

Immunocytochemical staining was processed in cell coverslips. Before fixation, coverslips were washed once with PBS. Then, cells were fixed by 4% paraformaldehyde for 10 min, permeabilized with 0.2% Triton X-100 for 5 min, and blocked with 5% bovine serum albumin for 1 h. The samples were stained with primary rabbit anti-Ki67 (ab16667, Abcam, dilution1:250) overnight at 4°C and subsequently with secondary donkey anti-rabbit IgG (H+L) (ab150073, Abcam, dilution1:1000) at room temperature for 1 h. All staining was examined under fluorescence microscope after counterstaining with 4′,6-diamidino-2-phenylindole dihydrochloride (DAPI) (D9542, Sigma, US).

### 2.8. Periodic Acid-Schiff Staining

Periodic Acid-Schiff (PAS) staining was processed in 4 *μ*m paraffinized sections. Briefly, the sections were deparaffinized and rinsed with distilled water. And the sections were immersed in the periodate alcohol solution for 10 min. Then, Schiff stain was added on the sections. After 10 min, the sections were washed with distilled water for 5 min. The nucleus was stained with Mayer hematoxylin solution. After flushing with running water, they were dehydrated and were transparent and sealed. PAS stain was used to observe the pathological changes of renal tubular basement membrane in this study.

### 2.9. Cell Proliferation Assay

Cell proliferation assay was assessed by cell counting kit-8 (CCK-8, Yesen, Shanghai, China). The CCK-8 utilized highly water-soluble tetrazolium salt (WST) to assay. The WST-8 [2-(2-methoxy-4-nitrophenyl)-3-(4-nitrophenyl)-5-(2,4-disulfophenyl)-2H-tetrazolium, mono-sodium salt] produces a water-soluble formazan dye upon reduction in the presence of an electron mediator. Briefly, NRK-52E cells were seeded at an initial density of 5 × 10^3^ cells/mL in a 96-well plate. Cells were treated with hypoxic-like incubation and/or rTSG-6. CCK8 solution (10 *μ*l) was dripped into each well. Then the 96-well plate was put in the incubator for 1 h. The solution was measured with a microplate reader at a wavelength of 460 nm and the optical density value was recorded.

## 3. Statistical Analysis

All data were expressed as the mean ± standard deviation (SD) and analyzed by using SPSS 17.0 (SPSS Inc., Chicago, IL, USA). Differences among groups were assessed by one-way ANOVA followed by Dunnett's tests. The significant statistical difference was defined as P<0.05.

## 4. Results

### 4.1. Renal Function and Morphologic Change on Different Days after Ischemia

**Figure 1:**
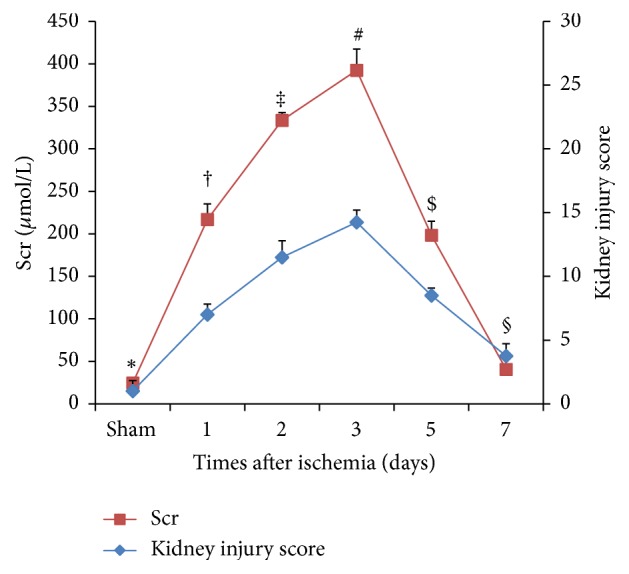
The Scr levels and kidney injury scores on different days after renal ischemia. The Scr levels (red diagram) and kidney injury score (blue diagram) on days 1, 2, 3, 5, and 7. Values presented are expressed as mean ± SD (n=6 in each group). Group with the symbol *∗* versus groups with symbols †, ‡, #, or $, P<0.05; group with the symbol *∗* versus group with the symbol §, P>0.05.

**Figure 2:**
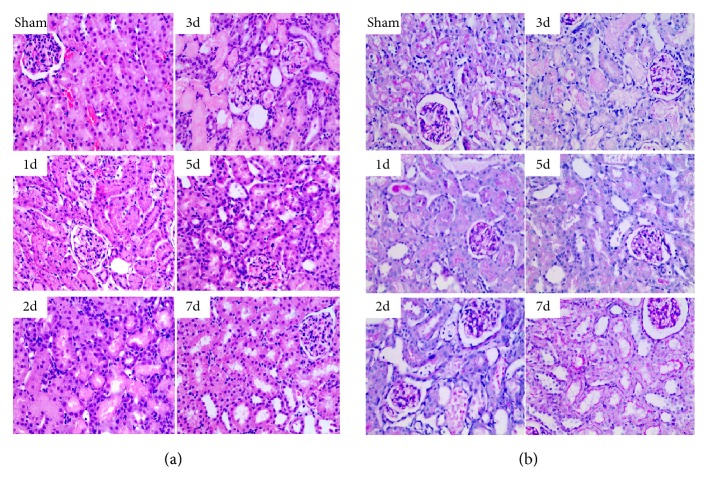
Renal histology on different days after renal ischemia. Changes in renal morphology at days 1, 2, 3, 5, and 7 ((a), H&E; (b), PAS) (original magnification 400×).

Rats were exposed to 45 min of bilateral renal ischemia and sacrificed on days 1, 2, 3, 5, and 7. After ischemia, rats showed marked deterioration of renal function with an increase in Scr levels on the 1st day (217.0±18.4 *μ*mol/L), 2nd day (333.5±9.2 *μ*mol/L), 3rd day (392.5±24.8 *μ*mol/L), and 5th day (198.4±16.5 *μ*mol/L) compared to basal levels of sham-operated rats (24.5±4.9 *μ*mol/L, all P<0.05). Furthermore, Scr reached its peak level on day 3. At 7 days, the Scr level (40.5±13.4 *μ*mol/L) was a little higher than the one in sham-operated rats, but there was no significant difference (Figure 1). Light microscopy of H&E-stained and PAS-stained kidney sections showed varying degrees of tubular epithelial cell necrosis, naked basement membranes, and tubular dilation with proteinaceous or cellular casts on days 1, 2, 3, and 5 after ischemia (Figures 2(a) and 2(b)). By day 7 after ischemia, renal histology was near normal (Figures 2(a) and 2(b)). Moreover, the kidney injury score was the highest on day 3 after ischemia (Figure 1). Accordingly, the third day was recognized as an observation point for subsequent studies.

### 4.2. Intravenous Transplantation of BMSC Attenuates IRI-AKI

**Figure 3:**
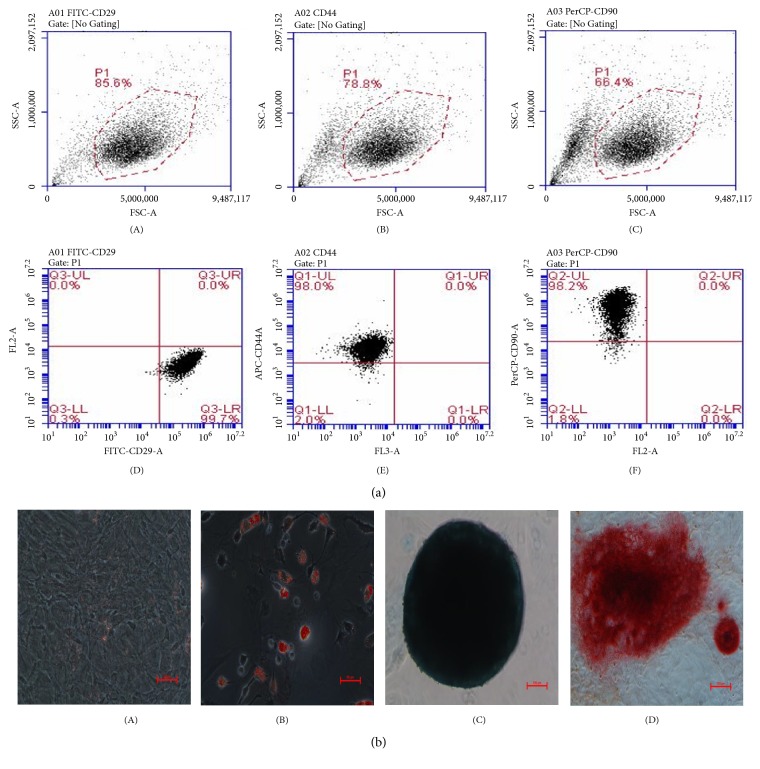
Identification of rat BMSC. (a) The typical markers CD29 (A, D), CD44 (B, E), and CD90 (C, F) of BMSC detected by flow cytometry. (b) Differentiation of BMSC (A, scale bar=50 *μ*m) into adipogenic (B, scale bar=50 *μ*m), chondrogenic (C, scale bar=100 *μ*m), and osteogenic (D, scale bar=100 *μ*m) lineages.

To identify rat BMSC, their typical surface markers and ability to differentiate were tested. Flow cytometric analysis confirmed that CD29, CD44, and CD90 surface markers in BMSC were positive (Figure 3(a)). The cell matrix exhibited fat drops in some cell bodies following oil red staining (Figure 3(b)-(B)), mucopolysaccharide deposition following alcian blue staining after 2-week induction (Figure 3(b)-(C)), and calcium deposition following the alizarin red staining (Figure 3(b)-(D)). These suggested that the BMSC had the ability to differentiate into adipocytes, chondrocytes, and osteoblasts.

**Figure 4:**
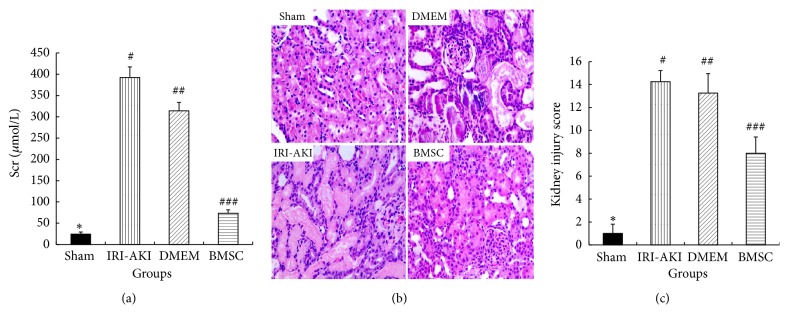
Scr levels and renal histology at 3 days after renal ischemia in IRI-AKI rats with different treatments. A significant decrease in Scr levels (a) was found in BMSC group compared to those in IRI-AKI group or DMEM group. Renal pathological lesions (b, c) in BMSC group were alleviated. (b) H&E staining, original magnification 400×. (c) Kidney injury score. Group with symbol *∗* versus other three groups with symbols #, ##, or ###, all P<0.05; group with symbol ### versus groups with symbol # or ##, both P<0.05; group with symbol # versus group with symbol ##, P>0.05.

To assess the therapeutic effect of BMSC on AKI, IRI-AKI rats were treated with BMSC or medium control (DMEM) intravenously. As shown in Figure 4(a), the Scr levels of IRI-AKI rats treated with BMSC (73.5±7.8 *μ*mol/L) significantly decreased compared to the IRI-AKI rats treated without BMSC (392.5±24.8 *μ*mol/L, P<0.05) or with DMEM (314.0±19.8 *μ*mol/L, P<0.05) at 3 days after 45-minute renal ischemia. Meanwhile, the renal tissue injury in BMSC group improved markedly compared to that in IRI-AKI group or DMEM group (Figures 4(b) and 4(c)). These suggested that intravenous transplantation of BMSC could attenuate IRI-AKI in rats.

### 4.3. The Therapeutic Effect of TSG-6-Silenced BMSC Weakened

**Figure 5:**
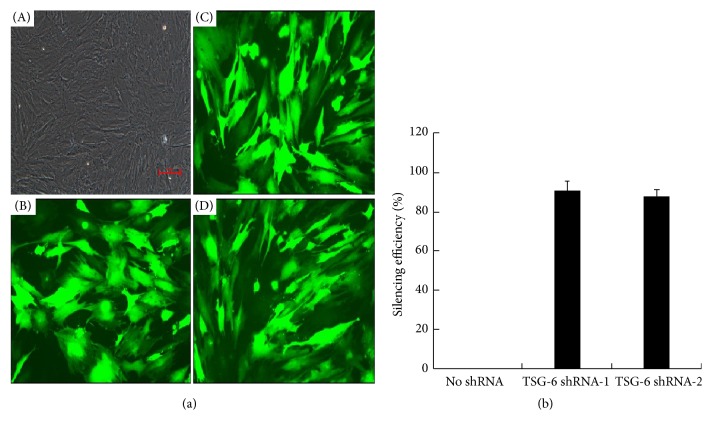
Assessments of transfection efficiency and silencing efficiency of TSG-6 shRNA. (a) BMSC transfected with shRNA were observed under bright field (A) and confocal microscope (B, scrambled control shRNA; C, TSG-6 shRNA-1; D, TSG-6 shRNA-2) (original magnification 200×). (b) BMSC were transfected with 50 *μ*L TSG-6 shRNA-1 or TSG-6 shRNA-2. Real time-PCR was used to quantify the mRNA expression of TSG-6. Silencing efficiency of no shRNA was defined as 0%. Silencing efficiency of TSG-6 shRNA= [1- amount of TSG-6 mRNA in BMSC transfected with TSG-6 shRNA/ amount of TSG-6 mRNA in BMSC without transfections] (%). Results (mean± SD) are from 3 sets of experiments.

To verify that TSG-6 plays a key role in the kidney protective function of BMSC, the BMSC were transfected with lentiviral vectors of TSG-6 shRNA to silence TSG-6. As shown in Figure 5(a), the efficiencies of TSG-6 shRNAs (TSG-6 shRNA-1 and TSG-6 shRNA-2) transfected into BMSC were more than 95%. The silencing efficiencies of the two TSG-6 shRNAs in BMSC were 90.6±4.9% and 87.5±3.5%, respectively (Figure 5(b)). Then, TSG-6 shRNA-1 was used for the subsequent study.

**Figure 6:**
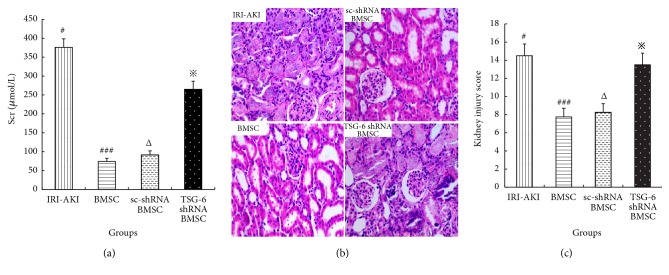
Scr levels and renal histology at 3 days after renal ischemia in IRI-AKI rats treated with BMSC after different pretreatments. A significant increase in Scr levels (a) was found in IRI-AKI rats treated with TSG-6 silenced-BMSC. Renal pathological lesions (b, c) in IRI-AKI rats treated with TSG-6 silenced-BMSC were more severe. (b) H&E staining, original magnification 400×. (c) Kidney injury score. Group with symbol *※* versus groups with symbol ### or Δ, both P<0.05.

As shown in Figure 6(a), the Scr levels of IRI-AKI rats treated with TSG-6-silenced BMSC (265.1±21.2 *μ*mol/L) significantly increased compared to the levels in IRI-AKI rats treated with BMSC (74.0±8.5 *μ*mol/L, P<0.05) or scrambled control silenced BMSC (91.5±10.6 *μ*mol/L, P<0.05) at 3 days after 45-minute renal ischemia. Meanwhile, the renal tissue injury in TSG-6 shRNA BMSC group worsened compared to those in BMSC group or sc-shRNA BMSC group (Figures 6(b) and 6(c)). These suggested that BMSC's therapeutic effect on IRI-AKI was weakened by silencing their TSG-6.

### 4.4. TSG-6 Promoted Renal Tubular Epithelial Cells to Proliferate

**Figure 7:**
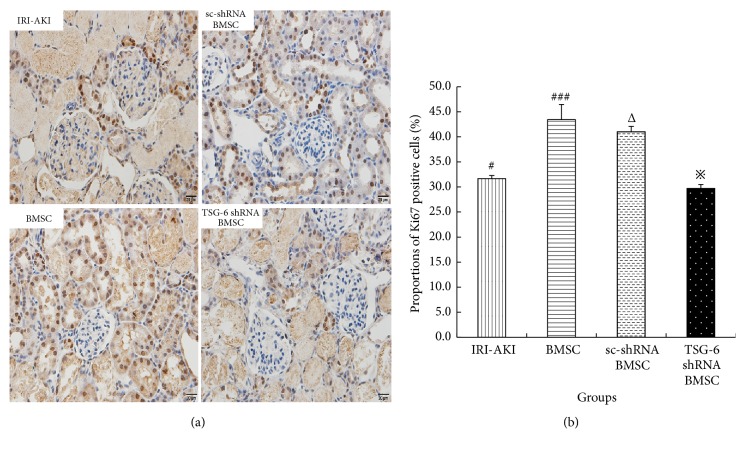
The proportions of Ki67-positive renal tubular epithelial cells in IRI-AKI rats treated with BMSC after different pretreatments on 3 days. (a) Ki67 immunostaining, original magnification 400×. The cells with nuclei stained brown were Ki67-positive cells. (b) The proportions of Ki67-positive cells in IRI-AKI rats treated with BMSC. Group with symbol # versus groups with symbol ### or Δ, both P<0.05; group with symbol *※* versus groups with symbol ### or Δ, both P<0.05.

To evaluate whether TSG-6 promotes renal tubular epithelial cells to proliferate, Ki67 immunostaining was performed. In IRI-AKI rats treated with BMSC, the proportion of Ki67-positive cells increased significantly compared to that in IRI-AKI rats (43.4±3.0% versus 31.7±0.6%, P<0.05). However, the proportion of Ki67-positive cells decreased significantly in IRI-AKI rats treated with TSG-6-silenced BMSC compared to that in IRI-AKI rats treated with BMSC (29.7±0.8% versus 43.4±3.0%, P<0.05) (Figures 7(a) and 7(b)). It suggested that TSG-6 promotes renal tubular epithelial cells to proliferate in IRI-AKI rats.

**Figure 8:**
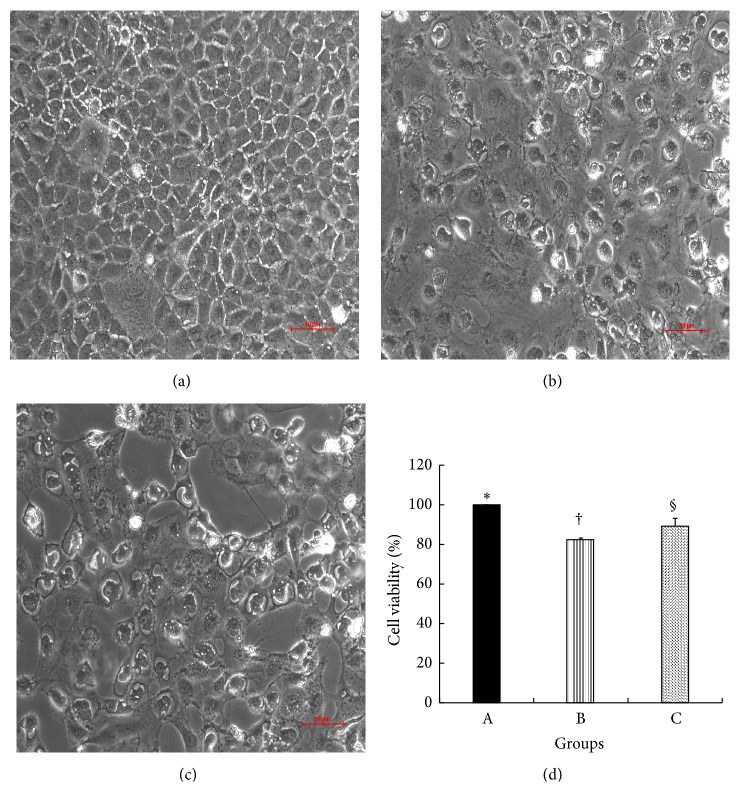
Morphological changes and cell proliferation rates of NRK-52E cells under hypoxia. (a~c) NRK-52E cells were observed under inverted microscope. Scale bar = 50 *μ*m. (a) NRK-52E cells under normoxia; (b) NRK-52E cells under hypoxia; (c) TSG-6-stimulated NRK-52E cells under hypoxia. (d) NRK-52E cells proliferation rates in groups A, B, and C. Group with symbol § versus group with symbol †, P<0.05. Results (mean ± SD) are from 6 sets of experiments.

**Figure 9:**
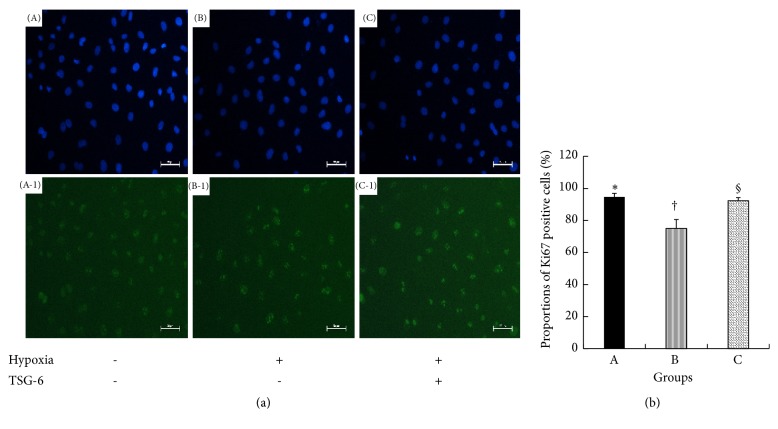
The proportions of Ki67-positive NRK-52E cells in groups. (a) DAPI nuclear staining (A, B, and C) and Ki67 immunostaining (A-1, B-1, and C-1), respectively. NRK-52E cells were observed under fluorescence microscope with different wavelengths of exciting light in the same field. Scale bar = 50*μ*m. (A, A-1) NRK-52E cells under normoxia; (B, B-1) NRK-52E cells under hypoxia; (C, C-1) TSG-6-stimulated NRK-52E cells under hypoxia. The cells with nuclei stained green were Ki67-positive cells. (b) The proportions of Ki67-positive cells in groups A, B, and C. Group with symbol § versus group with symbol †, P<0.05. Results (mean± SD) are from 6 sets of experiments.

To explore the effect of TSG-6 on proliferation of NRK-52E cells under hypoxia, cell proliferation assay analysis was utilized. It demonstrated that the cell proliferation rate of TSG-6 stimulated NRK-52E cells under hypoxia was 89.2±3.9%, which increased significantly compared to that of NRK-52E cells under hypoxia (82.4±0.8%, P<0.05) (Figure 8). Meanwhile, Ki67 immunostaining was performed on NRK-52E cells. In TSG-6 stimulated NRK-52E cells under hypoxia, the proportion of Ki67-positive cells (92.3±1.96%) was elevated compared to that in NRK-52E cells under hypoxia (75.0±5.6%, P<0.05) (Figure 9).

### 4.5. TSG-6 Inhibits Infiltration of Neutrophils in Kidney Tissue of IRI-AKI Rats

**Figure 10:**
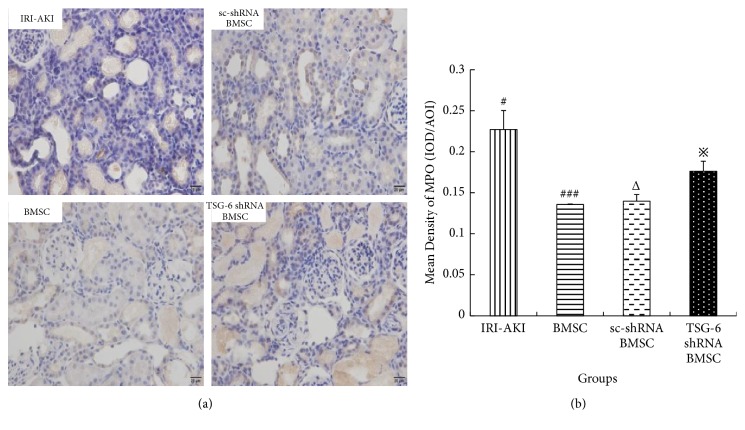
The mean density of MPO in kidney tissue of IRI-AKI rats treated with BMSC after different pretreatments on 3 days. (a) Immunohistochemical staining of MPO, original magnification 400×. (b) The mean density of MPO. Mean density = IOD/AOI (IOD, integrated optical density; AOI, area of interest). Group with symbol # versus groups with symbol ### or Δ, both P<0.05; group with symbol *※* versus groups with symbol ### or Δ, both P<0.05.

To explore the anti-inflammatory effect of TSG-6 in IRI-AKI, the levels of expression of MPO protein in kidney were determined by quantitative immunohistochemistry (Q-IHC) (Figures 10(a) and 10(b)). It showed that the level of expression of MPO in IRI-AKI rats treated with TSG-6 shRNA BMSC was significantly higher than those in IRI-AKI rats treated with BMSC or sc-shRNA BMSC (P<0.05). This indicated that TSG-6 could inhibit infiltration of neutrophils in kidney tissue of IRI-AKI rats.

## 5. Discussion

This study investigated the mechanism of BMSC ameliorating IRI-AKI in rats. The present study first illustrated that TSG-6 was the key factor in BMSC which ameliorated IRI-AKI in rats. Moreover, we found that TSG-6 worked through promoting renal proximal tubular epithelial cells to proliferate* in vivo* and* in vitro*.

At present, MSC is an ideal target cell for stem cell-based therapy. MSC has the ability to regulate immune response. Previous studies suggested that MSC could ameliorate kidney damage induced by IRI [12]. Also in our study, administration of BMSC intravenously resulted in significant improvements of renal function and renal histology, which was consistent with previous studies. However, the underlying mechanism of MSC's therapeutic effect is still unclear. It was found that when MSC were administrated into animal models of AKI, only a small proportion (0.1%~2.5%) of MSC could colonize and integrate into the damaged kidney [13]. Instead, the mechanism of MSC's effect is largely attributed to their unique anti-inflammatory and immune-modulatory properties via paracrine effects [14]. Among the secretory factors of activated MSC, TSG-6 has a powerful anti-inflammatory effect [7]. Its main functions include immunosuppressive modulation and extracellular matrix remodeling [15]. In particular, TSG-6 is used as a biomarker to predict efficacy of MSC in modulating sterile inflammation [16].

Previous studies have shown that the TSG-6 secreted by MSC is closely related to its therapeutic effects in several models of acute organs injury [17–20]. Lee et al. reported that TSG-6 knockdown in MSC could largely negate the heart protective function of MSC [17]. Similarly, Danchuk et al. proved that the beneficial effects of MSCs on LPS-induced lung injury were partly explained by the secretion of TSG-6 by MSCs [18]. Although TSG-6 has immunomodulatory effects on several inflammatory diseases, the factors secreted by MSC have different effects in different environments. Currently, there are few studies about the effect of TSG-6 secreted by MSC on kidney injury. One previous study showed that BMSC might exert the effects of anti-inflammation and antifibrosis on renal tubular cells under albumin-overloaded conditions via hepatocyte growth factor (HGF) and TSG-6 [21]. However, there was a lack of studies in IRI-AKI. In the present study, stable animal models of IRI-AKI were constructed successfully. And our data showed that the levels of serum creatinine reached a peak at 3 days after renal ischemia, which was consistent with previous reports [22]. Then, the third day was chosen as the observational point for subsequent study. Furthermore, the therapeutic effects of BMSC on IRI-AKI were largely abrogated by silencing their TSG-6. Therefore, TSG-6 was the key factor in BMSC which ameliorated IRI-AKI in rats.

Our study found that TSG-6 promoted renal tubular epithelial cells to proliferate in IRI-AKI rats. Moreover, our* in vitro* data showed that TSG-6 had promoted the proliferation of rat renal proximal tubular epithelial cells under hypoxia. These findings suggested that TSG-6 exerts its effect by promoting renal tubular epithelial cells proliferation. However, the underlying molecular mechanism remains unknown. It was reported that, in animal model of acute liver injury, TSG-6 could promote liver regeneration by suppressing inflammation [23]. Several previous studies showed that TSG-6 was an inflammation-associated secreted protein that had been implicated as having important and diverse tissue protections. Accordingly, in the setting of IRI-AKI, the proliferative effect of TSG-6 might be exerted through modulating inflammation. In the present study, we observed that TSG-6 could inhibit infiltration of neutrophils in kidney tissue of IRI-AKI rats. This might indicate that TSG-6 contributed to promoting renal tubular epithelial cells proliferation through its anti-inflammatory effect. In addition, several studies indicated that MSC could promote normal macrophages to transit into M2 phenotype [24]. Therefore, further studies will be needed to identify the underlying mechanism of TSG-6 to promote proliferation and anti-inflammatory effect in AKI.

In summary, our data suggest that TSG-6 is the key factor that allows BMSC to treat IRI-AKI. Furthermore, TSG-6 might exert its effects on promoting renal tubular epithelial cells proliferation by modulating inflammation. This study not only reveals the possible mechanism of BMSC treating AKI but also provides a novel drug (TSG-6) as an alternative to BMSC therapy.

## Data Availability

The articles used to support the findings of this study are included within the article and are cited at relevant places within the text as references.

## Abbreviations

MSC: Mesenchymal stromal cells
BMSC: Bone marrow derived mesenchymal stromal cells
IRI-AKI: Ischemia/reperfusion injury-induced acute kidney injury
TSG-6: Tumor necrosis factor-inducible gene 6 protein
shRNA: Short hairpin RNA
Scr: Serum creatinine
AKI: Acute kidney injury
CKD: Chronic kidney disease
IRI: Ischemia/reperfusion injury
SD: Sprague-Dawley
DMEM: Dulbecco modified Eagle's medium
PBS: Phosphate-buffered saline
FBS: Fetal bovine serum
CoCl_2_: Cobalt dichloride
rTSG-6: Recombinant human TSG-6
TSG-6 shRNA: Short hairpin RNA (shRNA) targeting rat TSG-6 gene
sc-shRNA: Scrambled control shRNA
GFP: Green fluorescent protein
MPO: Myeloperoxidase
H&E: Hematoxylin and eosin
DAPI: 4′, 6-Diamidino-2-phenylindole dihydrochloride
PAS: Periodic Acid-Schiff
CCK-8: Cell counting kit-8
WST: Water-soluble tetrazolium salt
IOD: Integrated optical density
AOI: Area of interest.
